# Why Plants Harbor Complex Endophytic Fungal Communities: Insights From Perennial Bunchgrass *Stipagrostis sabulicola* in the Namib Sand Sea

**DOI:** 10.3389/fmicb.2021.691584

**Published:** 2021-06-08

**Authors:** Anthony J. Wenndt, Sarah E. Evans, Anne D. van Diepeningen, J. Robert Logan, Peter J. Jacobson, Mary K. Seely, Kathryn M. Jacobson

**Affiliations:** ^1^Plant Pathology and Plant-Microbe Biology, School of Integrative Plant Sciences, Cornell University, Ithaca, NY, United States; ^2^Department of Integrative Biology, Michigan State University, East Lansing, MI, United States; ^3^W.K. Kellogg Biological Station, Michigan State University, Hickory Corners, MI, United States; ^4^B.U. Biointeractions and Plant Health, Wageningen Plant Research, Wageningen University and Research, Wageningen, Netherlands; ^5^Department of Biology, Grinnell College, Grinnell, IA, United States; ^6^Desert Research Foundation of Namibia, Windhoek, Namibia

**Keywords:** endophyte, latent saprophyte, decomposition, fungal community, drylands, nutrient islands

## Abstract

All perennial plants harbor diverse endophytic fungal communities, but why they tolerate these complex asymptomatic symbioses is unknown. Using a multi-pronged approach, we conclusively found that a dryland grass supports endophyte communities comprised predominantly of latent saprophytes that can enhance localized nutrient recycling after senescence. A perennial bunchgrass, *Stipagrostis sabulicola*, which persists along a gradient of extreme abiotic stress in the hyper-arid Namib Sand Sea, was the focal point of our study. Living tillers yielded 20 fungal endophyte taxa, 80% of which decomposed host litter during a 28-day laboratory decomposition assay. During a 6-month field experiment, tillers with endophytes decomposed twice as fast as sterilized tillers, consistent with the laboratory assay. Furthermore, profiling the community active during decomposition using next-generation sequencing revealed that 59–70% of the *S. sabulicola* endophyte community is comprised of latent saprophytes, and these dual-niche fungi still constitute a large proportion (58–62%) of the litter community more than a year after senescence. This study provides multiple lines of evidence that the fungal communities that initiate decomposition of standing litter develop in living plants, thus providing a plausible explanation for why plants harbor complex endophyte communities. Using frequent overnight non-rainfall moisture events (fog, dew, high humidity), these latent saprophytes can initiate decomposition of standing litter immediately after tiller senescence, thus maximizing the likelihood that plant-bound nutrients are recycled *in situ* and contribute to the nutrient island effect that is prevalent in drylands.

## Introduction

All perennial plants sampled to date harbor asymptomatic fungal endophytes in the apoplast of their stems, leaves and roots. Even in the most extreme habitats, where plant production and reproduction are severely constrained by thermal, moisture and/or nutrient stress, plants support diverse endophytic fungal communities (Rosa et al., 2009; Sun et al., 2012; Zabalgogeazcoa et al., 2013). Co-adaptive one-to-one relationships are well-known between endophytes and their plant hosts: fungi play roles in ameliorating thermal, drought, soil disturbance and herbivory-related stresses (Porras-Alfaro and Bayman, 2011), offsetting the nutritional costs associated with supporting specific endosymbionts. The question remains, however, why many plants support diverse endophyte communities instead of more specialized relationships to avail these services.

**FIGURE 1:**
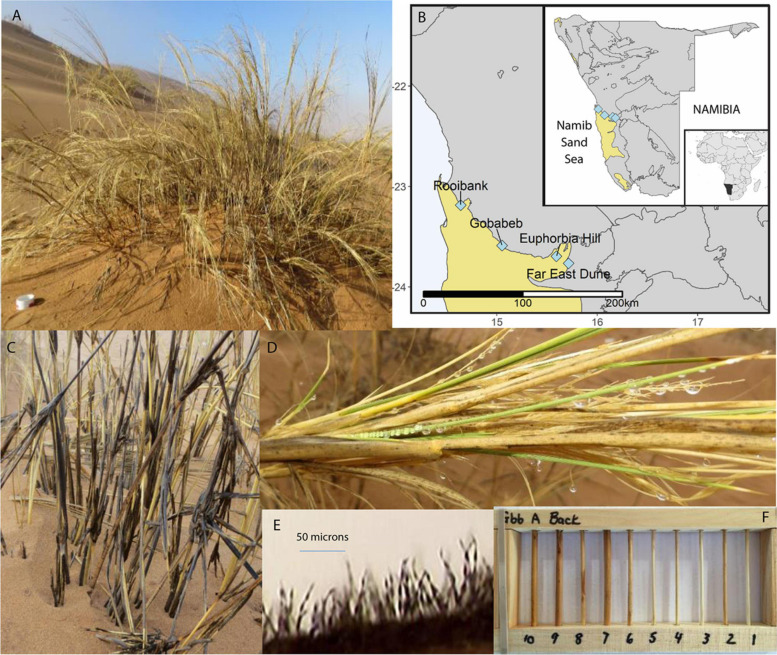
Overview of *Stipagrostis sabulicola* in the Namib Sand Sea (NSS) context. **(A)** *S. sabulicola* in the NSS. Scale is 10 cm diameter PVC pipe in the foreground. **(B)** Endophyte sampling sites spanning the width of the NSS. Maps created using public domain shapefiles accessed through the Natural Earth database (https://www.naturalearthdata.com/) and the Digital Atlas of Namibia (http://www.uni-koeln.de/sfb389/e/e1/download/atlas_namibia/main_ namibia_atlas.html). **(C)** Standing senescent tillers of *S. sabulicola* moistened by an 8-h fog are dark gray because of fungal growth in response to these frequent brief moisture pulses. **(D)** Dew droplets on living tillers and inflorescence. Note gray spots on senesced leaves which are fungal colonies. **(E)** Sporulating fungal hyphae on tiller surface following exposure to 10 h of 100% humidity. **(F)** Wooden racks containing 9-cm tiller sections used during the 6-month decomposition experiment at Gobabeb.

Given the energetic costs associated with supporting endosymbionts in general, and the potential challenges of navigating relationships with multiple endophytic taxa, it is still unclear what benefits might accrue to plants from hosting diverse endophytic communities. While these dynamics have been relatively unexplored among endophytes, associations between mycorrhizal fungi and their plant hosts offer a useful parallel. Mycorrhizae are similarly common associations between fungi and host plants that share nutritional and stress-related benefits, but under extreme abiotic stress (disturbance, drought, and salinity), plants may reject their mycorrhizal fungal partners (Allen, 1991). For instance, perennial grasses in the Namib Sand Sea (NSS; Figure 1) co-occur with their mycorrhizal fungi across a gradient of moisture stress, but drop these associations in the hyper-arid western regions of the NSS (Jacobson, 1997). To explore whether there are similar limits for plants supporting their endophytic symbionts, we sampled tillers of an endemic perennial grass, *Stipagrostis sabulicola* across this NSS gradient. We predicted that due to the moisture gradient, endophytic communities in the eastern region (∼65 mm/year) would be comparable to those in other drylands (Rosa et al., 2009; Sun et al., 2012; Massimo et al., 2015), but absent in the extreme environment of the western NSS (< 19mm/year) where plants appear to minimize their energetically costly interactions with microbial symbionts.

We also explored the benefits an endophyte community might afford its perennial host. We hypothesized that plants tolerate complex endophyte communities because they serve as latent saprophytes in standing tillers, initiating the decomposition process immediately after a tiller senesces. Endophyte communities are commonly comprised of fungi that are plant litter and soil saprophytes (Osono, 2006; Purahong and Hyde, 2011), and indeed, more than half of the saprophytic fungi isolated from standing litter of *S. sabulicola* in a previous study were common endophytes of plants in other ecosystems (Jacobson et al., 2015). Aridity does not preclude surface litter decomposition because saprophytic fungi are active during frequent non-rainfall moisture (NRM; i.e., fog, dew, and high humidity) and small rain events in the Namib (Evans et al., 2020). Indeed, a recent study revealed that community richness and Shannon diversity indices of litter fungi did not differ among eastern and western NSS sites, although the communities themselves were distinct (Logan et al., 2021). Furthermore, NRM is an important driver of surface litter decomposition in all grasslands studied to date: arid, semi-arid, and temperate grasslands and wetlands (Kuehn et al., 1999; Dirks et al., 2010; Wang et al., 2017; Evans et al., 2020; Lodato et al., 2021). Decomposition of grass tillers and leaves comprised of lignocellulose is a complex successional process accomplished by a suite of fungal species (Deacon, 2006; Lodato et al., 2021). If much of a plant’s endophyte community is comprised of latent saprophytes, this could explain why plants support diverse fungal communities, in addition to specific taxa that might support physiological processes of the living plant. Essential macronutrients needed for plant growth are notoriously low in dryland soils (Klopatek and Stock, 1994; Abrams et al., 1997; André et al., 1997; Jacobson, 1997), and therefore a perennial plant’s ability to efficiently recycle plant-bound nutrients from above-ground structures back to roots may be an essential trait ensuring its success. Indeed, nutrient islands surrounding perennial plants are a common feature of dryland plant communities (Schlesinger et al., 1995).

We used a multi-pronged approach to test our hypotheses. We determined the endophyte contribution to decomposition of freshly senesced standing litter with a field study of mass loss rates from sterile and non-sterile tillers over a 6-month period, predicting that if tillers contain endophytes that are latent saprophytes, sterilized tillers would decompose more slowly. We also assessed whether endophytes isolated from *S. sabulicola* were efficient decomposers *in vitro* and compared results of this experiment with the field study. Finally, we used next-generation sequencing (NGS) methods to estimate the overlap between living plant endophyte and year-old litter saprophyte communities. If plants support a diverse endophyte community because these fungi are an important inoculum source that initiates efficient standing litter decomposition immediately following senescence, we would expect a large proportion of the endophyte community to remain present and active in the decomposing surface litter.

## Materials and Methods

### Sites and Endophyte Sampling

Living tiller and dead tiller (litter) *S. sabulicola* samples originated from two regions, which span the moisture gradient of the NSS (Figure 1). NRM sources predominate at the two western sites: Rooibank (RB: S 23.186566° E 14.640453°) and Gobabeb (GB: S 23.588148° E 15.052270°) (Evans et al., 2020; Logan et al., 2021). In contrast, rainfall is the predominant moisture source at the two eastern sites: Euphorbia Hill (EH: S 23.694694°, E 15.593583°) and Far East Dune (FE: S 23.765639°, E 15.714639°). At Gobabeb, mean annual moisture derived from NRM is three times greater than mean annual rainfall (∼52 mm vs. ∼19 mm respectively) (Lancaster et al., 1984; Eckardt et al., 2013) whereas at the Far East Dune, mean annual rainfall is ∼65 mm and NRM is ten times less frequent than at Gobabeb (Evans et al., 2020; Logan et al., 2021).

Tiller samples were collected in January 2014 from asymptomatic living plants, with no visible fungal infection (western region: *n* = 5 (GB:2, RB:3); eastern region: *n* = 3 (FE:2, EH:1). Each sample consisted of at least ten tillers (4–5 mm diameter stems) from a single grass hummock (1–3 m diameter) located at least 20 m apart from other sample positions at the site. Tillers were cut to 20 cm lengths with a sterile razorblade and stored at 4°C in sterile Whirlpak plastic bags until fungal or DNA isolation was performed.

### Characterization of *S. sabulicola* Fungal Endophyte Community

#### Fungal Isolation

Endophytes were isolated according to a protocol adapted from Kumar and Kaushik (2013) and (Arnold et al., 2007). Twelve 15 mm-tiller segments from each sample were surface-sterilized, by submersion for 2 min each in 0.5% sodium hypochlorite followed by 70% ethanol, followed by a sterile water rinse. The effectiveness of the sterilization procedure was confirmed by imprinting on nutrient media (Schulz et al., 1998), prior to plating the tiller segment onto 2% MEA nutrient medium supplemented with antibiotic streptomycin (30 ug/ml). Subcultures from the growing edge of each emerging colony were taken prior to sporulation, transferred to 2% MEA nutrient plates, and incubated at 25°C in a dark growth chamber. Pure isolates were generated by subsequent rounds of sub-culturing. We made replicate stock cultures from each of these purified isolates that were stored at 4°C for immediate use for DNA extraction and the decomposition assay, and for long-term storage at −80°C in the KJCC permanent culture collection at Grinnell College (KJCC#1166-1236).

#### Identification, Characterization, and Culture-Dependent Diversity Assessment

Isolates were initially grouped into morphologically distinct operational taxonomic units (OTUs) and identified to genus and species using available taxonomic resources. DNA was then extracted from all unique OTUs and multiple isolates of common OTUs (60 total) according to the E.Z.N.A.^®^, Fungal DNA Mini Kit Protocol for fresh or frozen specimens (OMEGA Bio-Tek 2011). DNA was quantified using fluorometry and portions of the ITS region and the 18S rDNA gene were amplified via polymerase chain reaction (PCR) with Platinum^®^, PCR SuperMix (Life Technologies) and primers ITS1 + ITS4 (ITS region) and NS5 + NS6 (18 S rDNA gene) respectively. PCR products were purified using the QIAquick^®^ PCR Purification Kit Protocol and sequenced on an ABI Prism 3730XL sequencing instrument using the ABI prism BigDyeTM terminator cycle sequence kit (Applied Biosystems, Foster City, CA, United States). The SeqMan sequence alignment and analysis software (Lasergene; DNASTAR, Madison, WI, United States) was used for sequence editing and alignment. Sequences were identified by BLAST to Genbank TYPE entries^1^ (Ko et al., 2011) and CBS^2^ databases, and positive identification was determined as coverage of >80% and identity match of >98.0% (Raja et al., 2017). Sequence data for all identified isolates is archived in GenBank (accessions MF038136-MF038191). We performed two-sample t-tests (Minitab Version 19) to determine whether measures of abundance (mean isolation frequency, mean number of isolates/sample) and richness of isolates at the western sites differed from those at the east sites. We used the Welch’s test (not assuming equal variances) to account for possible effects of differences in sample size between groups.

#### Metagenomic Comparative Assessment of Whole Communities in Living and Senesced Host Material

We used NGS to compare endophytic communities in living tillers and saprophytic communities in dead surface litter (6 plants, 3 samples per plant) collected at Gobabeb and Far East Dune sites In November 2014. DNA was extracted from subsets of the eight *S. sabulicola* tiller samples used in the culture-dependent studies described above. Isolations were done on tillers that were surface-sterilized as above, using a MoBio Powersoil DNA extraction kit. DNA was stored at −80°C until further analysis.

For saprophytic communities, we wanted to identify taxa not only *present* on dead tillers, but also *actively decomposing* litter (i.e., functioning as saprophytes). To do this, we incubated samples with Bromodeoxyuridine (BrdU) and separated active DNA using immunocapture. BrdU is an analog of thymidine, and therefore can be used to tag proliferating, active cells (Borneman, 1999; Goldfarb et al., 2011; McMahon et al., 2011). We incubated the 18 litter samples at 25°C in sterile falcon tubes for 36 h, while growing microorganisms incorporated BrdU molecules into replicating DNA. We chose 36 h because it captures the period when much of the growth and activity of microbes after rewetting is known to occur (Iovieno and Baath, 2008; Placella et al., 2012; Göransson et al., 2013), but short enough to avoid significant fungal turnover or use of BrdU as a substrate by other microbes. DNA was extracted immediately after BrDU incubations using MoBio PowerSoil DNA Isolation Kit (MoBio Laboratories, Solano, CA, United States). After extraction, antibody immunocapture was performed to isolate BrdU-labeled DNA from inactive DNA (McMahon et al., 2011), resulting in 18 DNA samples of active DNA and 18 samples of ‘total’ DNA from the litter samples. We evaluated the efficacy of the BrdU immunocapture by visualizing DNA and amplified PCR product on agarose gels, and by comparing bands to a control sample incubated with only water, without BrdU.

Fungi from both the dead litter (*n* = 18 active and 18 total) and living tillers (*n* = 8) samples were characterized by amplifying the ITS1 region using primers ITS1-F/ITS2 and sequencing on the Illumina MiSeq platform (Michigan State University Genomics Core Facility). Sequences were processed using the USEARCH pipeline (v.8.1). We identified representative sequences for each OTU with referenced (UNITE) based chimera checking, and made taxonomic assignments using utax (v. 9.2) classifier against UNITE 7.1. The OTU table was constructed using USEARCH with taxonomic assignments from RDP (Kõljalg et al., 2013). Sequencing resulted in an average of 31,032 sequences per sample, before filtering. We included three blanks that followed the same extraction protocol as the samples but that instead used sterile water. The 32 species reported in the blanks were removed from the 44 experimental samples. Filtering included exclusion of samples with sequence numbers < 1000, taxa that were not present in more than two samples of the whole dataset (endophyte & saprophyte), and taxa with less than 5 reads across endophyte samples (*n* = 8) and 25 reads across decomposer samples (*n* = 36). All sequences are available in NCBI under the BioProject ID of PRJNA726949.

To examine endophyte diversity, we rarefied samples to the lowest sequence per sample (∼18,000) and calculated mean richness per site. We were interested in the dual presence of OTUs in dead litter (acting as saprophytes) and surface-sterilized live stems (acting as endophytes), so we summed the sequence counts for all saprophyte samples within sites and quantified the occurrence of endophytes in the aggregated saprophytes. We also examined the relative abundance of these taxa in endophyte versus saprophyte samples, testing differences using the same statistical procedures as above.

### *In vitro* Decomposition Assays

Newly senesced standing *Stipagrostis sabulicola* tiller litter was collected from plants at the Gobabeb site in June 2014 for the decomposition assay. The flowering stalks of these tillers were still in place indicating recent senescence. Yellow tillers (4–5 mm diameter) with no signs of coloration, cuticle damage, or fungal infection, were cut with a sterile razor blade in the late afternoon when relative humidity was 10–20%, air-dried, and kept in sterile Whirlpak bags at room temperature until used.

The decomposition assay was based on a protocol developed by Osono and Takeda, 2006. Yellow tiller litter was cut to 10 mm lengths, halved laterally, and dried at 60°C overnight. Sets of three segments comprised each sample and were weighed, placed in microcentrifuge tubes and sterilized by dry autoclaving at 120°C for 20 min. Autoclaving did not affect the mass of the segments: dry mass of three randomly chosen samples before and after autoclaving did not differ (T-stat = 1.0, *p* = 0.423). Using sterile technique, the three sterile segments comprising each sample were placed approximately 10 mm apart and 15 mm from a designated central inoculation site on Petri dishes containing water agar. Subcultures (2 × 2 × 2 mm) from the growing margin of 66 endophyte isolates were placed on the central inoculation point of each Petri dish (*n* = 10). Controls were similarly prepared but without inoculation. The assay and control plates were incubated in a growth chamber for 28 days with a diel cycle of thermal and light regimes that simulated average summer ambient conditions at ∼ 30 cm above the sand surface on *S. sabulicola* tillers at Gobabeb (Ebner et al., 2011): 10 h - 15°C, dark; 2 h - 20°C light, 1 h - 25°C light; 1 h - 30°C, light; 4 h - 35°C, light, 2 h - 30°C, light; 2 h - 25°C, light; and 2 h - 20°C, light. At the conclusion of the incubation period, all tiller samples were brushed free of superficial mycelial growth using a dry task wiper (Kimberly-Clark^TM^ Professional Kimtech Science^TM^ Kimwipes^TM^), oven-dried at 60°C, and weighed to determine final dry mass.

Mean percent mass loss was computed for each fungal isolate as a measure of decomposition ability (Osono and Takeda, 2006). We determined that an isolate was able to decompose *S. sabulicola* tillers if mean percent mass loss was significantly greater (*p* < 0.05) than that of the un-inoculated controls, using 1-way 2-sample *t*-tests. Mean normalized decomposition ability (NDA) was then computed for each isolate by subtracting the mean percent mass loss of un-inoculated controls from percent mass loss of each isolate sample. Mean NDA for each isolate most accurately represents the percent mass loss attributable solely to the fungal treatments, by accounting for mass loss that occurred from leaching into the water agar medium or by photodegradation. A *t*-test was used to determine whether the mean percentage of saprophytic isolates (i.e., with positive NDA) differed between the western and eastern regions of the dune field.

Laccase and peroxidase enzyme assays (Viswanath et al., 2008) were conducted on all endophytic isolates. We predicted that as primary colonizers, these isolates would exhibit minimal lignin-degrading enzyme activity. We also performed a *post hoc* 2-sample *t*-test to determine whether there was a significant difference in the NDA of isolates based on presence or absences of lignin-degrading enzymes.

### Endophyte-Mediated Decomposition *in situ*

We deployed sterilized and un-sterilized newly senesced tillers in wooden racks 40 cm above the ground at Gobabeb for 6 months, to determine whether endophytes play a measurable role in standing tiller decomposition immediately following senescence (Figure 1C). Ambient moisture and temperature conditions for 15 June through 15 December 2016 were determined from data downloaded from the Gobabeb Met SASSCAL meteorological station^3^. Twenty tillers from Gobabeb (see decomposition assay above for details) were cut to standard 9 cm lengths, half were dry-autoclaved to eliminate the endogenous fungal community, and half were not. Each tiller was weighed using an analytical balance (to 0.1 mg) and loaded randomly into two wooden racks. After 6 months of exposure, the mass of each tiller was determined as above, and differences in percent mean mass loss between the endophyte and sterilized treatments were compared using two-sample *t*-tests.

Field mass loss resulting from the endophyte community was also compared with the range of NDA obtained for the isolates by converting both to hourly rates. The total number of hours of fungal activity during the field experiment was conservatively estimated by summing the number of hours during the 6-month period when relative humidity was greater than 90%, and lasted for at least 6 h (55 events, 457 h). Previous investigations have shown that saprophyte communities of S. sabulicola are metabolically active under these conditions (Jacobson et al., 2015; Evans et al., 2020). The total number of hours of fungal activity during the *in vitro* experiments was 672 h (28 days × 24 h), because the isolates were able to grow at all temperatures used in the diel cycle (Jacobson et al., 2015) and moisture was never a limiting factor.

## Results

### Richness and Abundance of Endophytes Associated With Living and Senesced *S. sabulicola* Tillers

**FIGURE 2:**
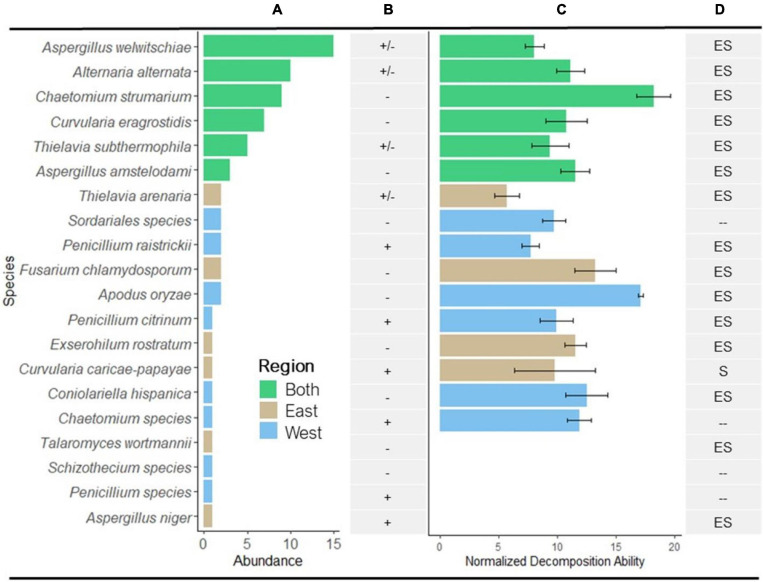
Abundance and decomposition ability of isolated fungi. **(A)** Distribution of the 20 identified species from eastern and western regions of the Namib Sand Sea (see Table 1 for number of isolates/species). Bar colors correspond to geographical regions. **(B)** Presence or absence of lignin-degrading enzymes laccase and peroxidase; (+), enzyme presence in all isolates; (–), no enzyme present; (±), enzyme present in some isolates and not present in others. **(C)** Mean normalized decomposition ability (NDA; mean % mass loss) of isolates that were able to decompose sterile *S. sabulicola* tillers over 28 days in the Namib diel cycle. Values were normalized relative to mass loss in un-inoculated tillers (Isolate sample size as in A, error bars = 1 × SE; *n* = 10 for each isolate). **(D)** Summary of known nutritional modes of endophytic species isolated from *S. sabulicola*: E, endophyte; S, saprophyte; –, no data as taxa was not identified to species (see Table 1 for references).

We obtained 71 endophyte isolates from 96 *S. sabulicola* tiller segments, corresponding to an isolation frequency across both regions of 74%. All but three of the 71 isolates were identified at the order level or lower using sequence data. These three had no morphologically identifiable features and were excluded from subsequent analyses. The remaining 68 isolates comprised 20 different taxa (Figure 2 and Table 1), of which four were identified at the generic level but could not be resolved to species. The sequences of these four taxa were aligned with congeneric taxa from the data set and were distinct, so were included in subsequent analyses.

**TABLE 1:** Endophytic taxa isolated from *Stipagrostis sabulicola*.

Taxon (order, family, genus, and species)	Coverage/similarity (%)	Laccase and peroxidase assay	Endophyte references	Saprophyte references
***Eurotiales***				
***Aspergillaceae***				
*Aspergillus amstelodami* Thom and Church	99/99	No (3/3)	Sanchel Marquez et al., 2008	Pinciroli, 2013
*Aspergillus niger* Tiegh	92/99	Yes (1/1)	Sanchel Marquez et al., 2008	Jacobson et al., 2015
*Aspergillus welwitschiae* (Bres.) Henn.	92/99	Yes (12/14)	Liu et al., 2019	Hong et al., 2013
*Penicillium citrinum* Thom	100/100	Yes (1/1)	Khan et al., 2008	Pinciroli, 2013
*Penicillium raistrickii* G. Sm.	100/100	Yes (2/2)	Busby et al., 2016	Pandey and Palni, 1998
*Penicillium* species	No match	Yes (1/1)	NA	NA
***Trichocomaceae***				
*Talaromyces wortmannii* (Kloeker) C.R. Benj.	100/98	No (1/1)	Bara et al., 2013	Yilmaz et al., 2014
***Hypocreales Nectriaceae***				
*Fusarium chlamydosporum* Wollenw and Reinking	100/100	No (2/2)	Ananda and Sridhar, 2002	Jacobson et al., 2015
***Sordariales***				
*Sordariales* species	No match	No (2/2)	NA	NA
***Chaetomiaceae***				
*Chaetomium strumarium* (Rai, Tew, and Muki) Cann.	98/99	No (10/10)	Uma Anitha and Mythili, 2017	Jacobson et al., 2015
*Chaetomium* species	No match	Yes (1/1)	NA	NA
*Thielavia arenaria* Mouch.	98/99	Yes (1/2)	Maciá-Vicente et al., 2008	Jacobson et al., 2015
*Thielavia subthermophila* Mouch.	99/99	Yes (2/5)	Mouchacca, 2007	Jacobson et al., 2015
***Lasiosphaeriaceae***				
*Apodus oryzae* Carolis and Arx	No TYPE; 99/99	No (2/2)	Angelini et al., 2012	Geydan et al., 2012
*Schizothecium* species	No match	No (2/2)	NA	NA
***Pleosporales Pleosporaceae***				
*Alternaria alternata* E.G. Simmons	98/99	Yes (6/10)	Kumar and Kaushik, 2013	Jacobson et al., 2015
*Curvularia caricae-papayae* H.P. Srivast. and Bilgrami	99/100	Yes (1/1)	Not known	Not known
*Curvularia eragrostidis* (Henn.) J.A. Mey.	98/99	No (6/6)	Kaushik et al., 2014	Granados-Montero et al., 2018
***Xylariales Xylariaceae***				
*Exserohilum rostratum* (Drechsler) K.J. Leonard and Suggs	99/98	No (1/1)	Costa et al., 2012	Gomaa et al., 2015
*Coniolariella hispanica* Checa, Arenal, and J.D. Rogers	100/99	No (1/1)	Checa et al., 2008	Hao et al., 2017

*NCBI Blast results % coverage and % Similarity are all TYPE search results unless otherwise indicated. Results of the laccase and peroxidase enzyme assays: *Yes* vs. *No* indicates whether isolates tested positive or negative in enzyme assay (with bracketed fraction indicating the proportion of the isolates tested). Summary of known nutritional modes for each taxon.*

Of the 20 identified taxa, the six most abundant species were found in both regions, collectively comprising 72% of all identified isolates: *Aspergillus welwitschiae* (15 isolates), *Alternaria alternata* (10), *Chaetomium strumarium* (9), *Curvularia eragrostidis* (7), *Thielavia subthermophila* (5), and *Aspergillus amstelodami* (3) (Figure 2A). Of the remaining 14 species (19 isolates), comprising 28% of the isolates, 12% (6 species, 8 isolates) were found only in the eastern region and 16% (8 species, 11 isolates) were found only in the western region. We observed no difference in mean richness and abundance between the eastern and western regions of the NSS (*p* = 0.921 and *p* = 0.414, respectively) (Table 2).

**TABLE 2:** Comparison of the endophyte communities in living tillers in the eastern and western regions of the Namib Sand Sea. Mean abundance, richness and decomposition metrics (± SD) of fungal endophyte communities of *S. sabulicola* obtained by (1) isolate-based sampling and (2) next-generation sequencing.

1. Isolate-based sampling				
	Region			
	West	East	T-stat	*p* value
Mean % isolation frequency (isolates/segment)	63 (9.5)	78 (5.2)	1.58	0.364
Mean isolates/site	19.5 (2.1)	14.5 (4.9)	1.31	0.414
Mean richness/site	9 (5.6)	8.5 (0.7)	0.12	0.921
% NDA + isolates/site^1^	64 (8)	45 (12)	0.75	0.590
Mean % mass loss	12.64 (4.78)	10.37 (3.91)	1.70	0.098

**2. Sequence-based sampling**				

	**Region**			
	**West**	**East**	**T-stat**	***p* value**

Mean OTU richness/site – all samples	37.6 (6.61)	38.8 (7.4)	0.19	0.854
Mean OTU richness/site – abundant^2^ samples only	22 (6.55)	27.2 (6.61)	1.082	0.335
Mean% *active* saprophytic richness in living tillers^3^	57.35 (8.14)	67.18 (9.87)	1.46	0.242
Mean% *active* saprophytic abundance in living tillers^4^	55.5 (21.4)	83.4 (23.6)	1.67	0.193

*^1^NDA + isolates are those capable of decomposing dead *S. sabulicola* tillers. ^2^Abundant defined as sum of occurrence across all 8 samples >20. ^3^OTUs present in living tillers that were also in active litter. ^4^Reads of OTUs present in living tillers that were also in active litter.*

Corroborating the results from the isolate-based approach, we observed no significant differences in richness between the eastern and western regions using sequence-based community profiling (Table 2). NGS sequencing identified 168 endophyte taxa associated with living tillers. This is much greater than the richness values obtained from the isolate-based community assessment, which was expected because previous studies have shown that very large numbers of isolate samples are needed to yield comparable richness assessments to high-throughput sequencing (O’Brien et al., 2005; Arnold et al., 2007; Higgins et al., 2011). Despite the differences in richness estimates from the two methods, both revealed communities with similar structure, comprised of a small number of abundant taxa and a large number of rare taxa: the 50 most abundant taxa (30% of taxa) comprised 96% of the read abundance (isolates: top 30% comprise 72%). A comparison of only the most abundant OTUs across the two regions confirm the insignificant results from the isolate-based study.

Furthermore, both the mean proportion of active saprophytic taxa (latent saprophytes) and their abundance in the living tillers were not significantly different in the east and west (*p* = 0.242, Table 2). Saprophytic function of latent saprophytes borne within the host plant does not differ at the sites. Notably, in both regions active saprophytic taxa make up a large proportion of endophytic communities, comprising 57–67% of the taxa that account for 56–83% of endophyte abundance.

**FIGURE 3:**
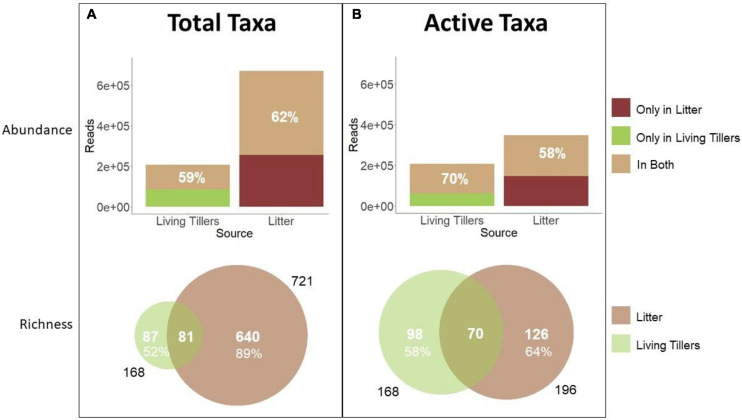
Abundance and richness of saprophytic fungi in living and senescent tillers. Comparison between the endophyte community and **(A)** the total litter community or **(B)** the active litter community. Sequence read abundance and OTU richness of latent saprophytes in living tillers (olive; *n* = 8 samples) and of saprophytes in dead litter (brown; *n* = 18 samples) is based on NGS data.

Total litter saprophyte community richness (721 taxa) was much greater than the living tiller endophyte community (168 taxa), in part because of differences in sampling effort (*n* = 18 vs. 8) (Figure 3). However, even at the sample level, mean richness was significantly greater in the total litter (119 ± 14) than in the living tillers (38 ± 3) (Table 3; *p* < 0.001). Likewise total read abundance of all litter samples was larger because of sampling effort differences, but even at the sample level, mean read abundance was 1.5× greater in the year-old litter samples (Table 3; *p* = 0.04). Other studies have similarly shown that saprophytic communities are more diverse than endophyte communities (Guerreiro et al., 2018). Overall, 81 latent saprophyte taxa (shared OTUs present in both living tillers and year-old gray litter) comprised 48% (81/168) of the endophyte (living tiller-derived) community richness, but only 11% (81/721) of the total saprophyte (litter-derived) community richness (Figure 3A). Despite these differences in overall richness, these shared taxa comprised roughly 3/5 of the read abundance in both endophyte and saprophyte communities (59 and 62%, respectively; Figure 3A), which did not differ significantly at the sample level (Table 3; shared richness: *p* = 0.172, shared abundance: *p* = 0.567). Thus, while the latent saprophyte taxa are proportionally abundant in the endophyte community prior to senescence, this relatively small number of latent saprophytic taxa (81/720) comprise a disproportionately larger fraction of the read abundance in the total litter (62%).

**TABLE 3:** Next-Generation Sequencing (NGS) data comparing living tillers and litter communities on a per sample basis.

Total litter						
		Mean	SE	*N*	T-value	*p*-value
*Richness*	Living tillers	38	3	8	5.84	< 0.001
	Total litter	119	14	18		
*Abundance*	Living tillers	25,878	4,166	8	2.28	0.04
	Total litter	37,188	2,685	18		
*Shared richness*	Living tillers	22	1	8	1.41	0.172
	Total litter	18	2	18		
*Shared abundance*	Living tillers	19,628	4,540	8	0.58	0.567
	Total litter	23,079	3,779	18		

**Active litter**						
		**Mean**	**SE**	***N***	***T-*value**	***p-*value**

*Richness*	Living tillers	38	3	8	0.54	0.588
	Active litter	35	2	18		
*Abundance*	Living tillers	25,878	4,166	8	1.21	0.243
	Active litter	19,367	3,385	18		
*Shared richness*	Living tillers	23	1	8	0.23	0.817
	Active litter	24	3	18		
*Shared abundance*	Living tillers	18,049	4,509	8	1.37	0.200
	Active litter	11,092	2,307	18		

While this result hints at the functional importance of the latent saprophytes in the litter community, we used BrDU analysis to explicitly quantify taxon activity. We first identified the active litter community after 36 h of wetting and then determined the proportion of latent saprophytes that were amongst these active taxa. Roughly 27% (196/721) of all the litter OTUs were active in the litter (Figure 3B), and a mean of 35 (± 2) OTUs were active in each litter sample (Table 3). Active latent saprophytes (70 shared OTUs) comprised 42% of the endophyte community richness, and 70% of the read abundance in living tillers prior to senescence (Figure 3B). The latent saprophytes are similarly abundant (58%) in the active litter samples a year post-senescence; and read abundance of living tillers and active litter on a per sample basis were not significantly different (Table 3; *p* = 0.200). Clearly, latent saprophytes comprise an important part of the functional saprophytic community in the litter more than a year post-senescence.

Thus, both NGS methodologies yielded similar outcomes: more than half (59–70%) of the *S. sabulicola* endophyte community is comprised of latent saprophytes, and these dual-niche fungi still comprise a similarly large proportion (58–62%) of the litter community more than a year after senescence.

### Decomposition Potential of *S. sabulicola* Endophyte Isolates

A literature survey revealed that all but one of the identified endophytic species have been previously reported as both endophytes and as saprophytes (Figure 2D and Table 1).

Furthermore, of the 20 taxa evaluated, 80% were capable of decomposing tillers during the 28-day *in vitro* decomposition assay (mean NDA = 11.14%; SD = 3.20, SE = 0.80) (Figure 2C). There was no relationship between mean NDA and relative abundance, although 4/6 of the most abundant species had mean NDA values greater than the overall mean. The range of mean NDA for isolates capable of decomposing litter material was 6.50–17.88%, or 0.97–2.66 × 10^–2^%/hour.

There was no significant difference in the proportion of isolates from the west (64%) and east (45%) that could decompose *S. sabulicola* tillers during the 28-day experiment, nor in the mean % mass loss of these isolates from the two locations (Table 2). Six saprophytic species occurred in both the western and eastern regions, four were unique to the east, and six were unique to the west (Figure 2C). This relatively even distribution of cosmopolitan and region-specific saprophytic endophytes (latent saprophytes) across the NSS suggests that endophytes capable of saprotrophy are equally prevalent in both regions. While the taxonomic composition of the endophyte communities may differ across regions, the overall saprophytic ability of the endophyte community is consistent across the study area.

**FIGURE 4:**
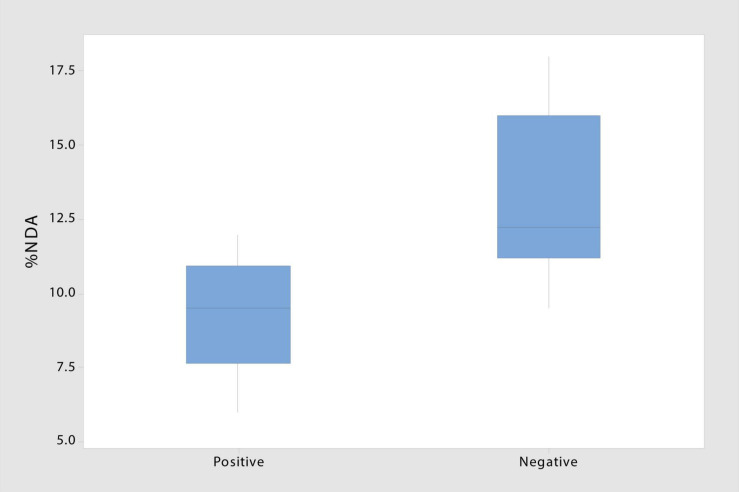
Decomposition ability of taxa testing negative for laccase and peroxidase enzymes involved in lignin degradation is significantly higher than that of taxa testing positive (T-stat = 3.09; *p* = 0.009) (*n* = 8).

The presence of laccase and peroxidase enzymes varied greatly across taxonomic groups and even within species (Figure 2B and Table 1). Two of the four taxa with insignificant NDA values tested negative for both enzymes, suggesting that these species (*T. wortmannii* and *Schizothecium spp.*) may lack saprophytic abilities and function strictly as endophytes. Of the 16 saprophytic taxa tested, half tested positive for lignin-degrading enzymes, but notably, isolates with no enzyme activity had significantly higher NDA (*p* = 0.009) than those that tested positive (Figure 4). While this result requires further study, a plausible interpretation is that high NDA (lignin-degrading enzymes absent) isolates are those that perform well as early colonizers competing for simpler cellulose moieties; whereas low NDA (enzymes present) isolates are specialized for later stages of decomposition when recalcitrant lignin becomes available (Frankland, 1976; Deacon, 2006). It is also plausible that the two taxa (*Penicillium sp.* and *Aspergillus niger*) that tested positive for lignin-degrading enzymes but had insignificant NDA values were simply not efficient enough to register in our brief decomposition assay. Furthermore, *A. niger* is known to be a particularly efficient degrader of tannins, and because tannins bind with proteins, Van Diepeningen et al. (2004) proposed that this fungus may play a unique role modulating nitrogen availability in litter communities. It thus appears that most of the endophytic taxa of *S. sabulicola* that we isolated are decomposers, and that this latent-saprophytic community is comprised of taxa showing specialization for different stages of the decomposition process.

### *In situ* Decomposition Ability of the *S. sabulicola* Endophyte Community

**FIGURE 5:**
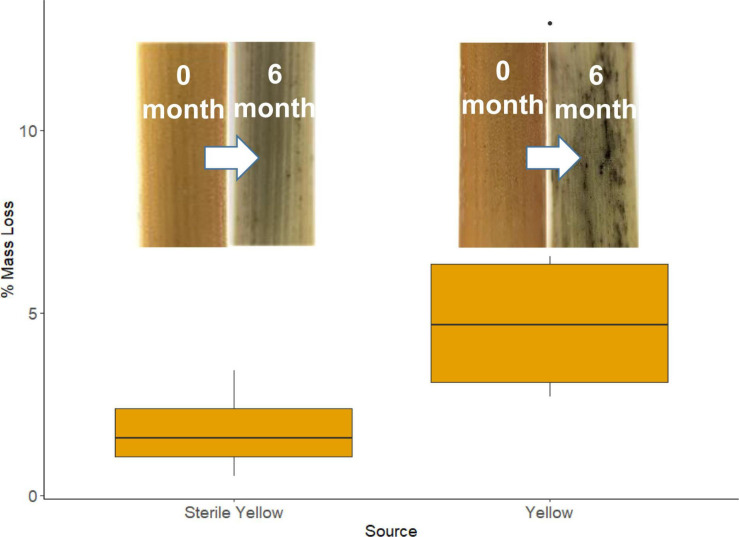
Mean percent mass loss from recently senesced sterile and non-sterile yellow *S. sabulicola* tillers. Inserts are of lower portions of sterile yellow tillers at 0 and 6 months, and unsterilized yellow tillers at 0 and 6 months.

During the 183-day period when tillers were in the field, there was no rainfall at the study site, but there were 55 nights that met our “wet” criteria, amounting to 457 h during which fungi could be metabolically active (Jacobson et al., 2015). The mean length of each event was 8.5 h (SE = 0.3). We observed significantly greater mass loss from unsterilized tillers containing endophytes compared to sterilized tillers: 5.00% (SE = 0.74) vs. 1.78% (SE = 0.31) (Figure 5; *p* = 0.002). The tiller mass loss rate of 1.2 × 10^2^%/hour from the unsterilized material provided a whole-community estimate of mass loss from recently senesced tillers which falls well within the range of mass loss rate for individual taxa (0.97–2.66 × 10^–2^%/hour) obtained via the *in vitro* decomposition assays. While a possible explanation for the smaller mass loss from the sterile material is that the sterilization process made the substrates unpalatable, it should be noted that dry-autoclaved litter was readily decomposed by fungal isolates in the *in vitro* decomposition trials, suggesting that sterilization did not alter the physical or chemical properties in a fashion that prevented fungi using them as substrates.

## Discussion

Our multi-pronged study yields strong evidence supporting the hypothesis that dryland plants tolerate complex endophyte communities in living tillers because these fungi initiate decomposition of standing tillers immediately post-senescence. Both NGS- and isolate-based sampling methods revealed that endophyte communities are abundant and species-rich in *S. sabulicola* stems throughout the NSS (Tables 2, 3 and Figures 2A, 3). This community plays a large role in actively decomposing litter post-senescence: litter with intact endophyte communities decomposed twice as fast over a 6-month period as sterile litter colonized solely by airborne fungal propagules (Figure 5). Furthermore, all but one of the taxa we isolated as endophytes are also known saprophytes of plant litter (Figure 2D and Table 1). This overlap in potential function is well known from endophyte studies in a variety of habitats (Promputtha et al., 2007; Su et al., 2010; Purahong and Hyde, 2011; Sun et al., 2011; Sánchez Márquez et al., 2012), but no studies have confirmed that specific endophyte isolates are functional saprophytes (Porras-Alfaro and Bayman, 2011; Vázquez de Aldana et al., 2013). Our decomposition assay conclusively showed that 80% of these endophyte taxa were efficient decomposers of their host tillers (Figure 2C), which is compelling evidence that the endophyte community is composed of “dual-niche” fungal genotypes that function as both saprophytes and endophytes in their host plants.

The decomposition assay also revealed inter- and intra-specific differences in degradation ability among endophytes (6.5–18%) (Figure 2C). This difference emerged even over our short assay period, which is just a mere snapshot of their ability to decompose newly senescent litter. An analysis of functional ability throughout the multi-year decomposition process was beyond the scope of this study, but we did find that isolates lacking lignin-degradation capabilities were more efficient decomposers than those that had laccase and peroxidase function (Figure 4). This suggests that the latent saprophytic communities that occupy living plants are functionally diverse, exhibiting trade-offs between strategies of efficient cellulose decomposition immediately post-senescence versus specialization for more recalcitrant lignin substrates, likely later in the decomposition process (Dix and Webster, 1995). Remarkably, once corrected for hours of wetness when fungi would be active, the decomposition rates measured for the community in the field fell well within the range of those measured for individual species in our short lab-based assay.

We also found that the latent saprophyte community remains an important part of the litter saprophyte community at least a year after senescence. Active taxa comprised roughly comprised roughly 40% of both the living tillers (42%) and the litter (36%) community richness (Figure 3B). Not surprisingly, these 70 taxa comprise a large share of the endophyte abundance in living tillers (70% of reads), but remarkably, also comprise almost 3/5 of the abundance in year-old gray litter (58%), despite opportunities for the senesced litter to be colonized by the 126 active litter saprophyte taxa present in the environment which do not colonize living tillers. Other recent studies have similarly found that plants harbor endophyte communities that include latent saprophytes (Baldrian et al., 2012; Szink et al., 2016; Guerreiro et al., 2018). In particular, Guerreiro et al. (2018) found that endophytes comprised a very large portion of the reads and about a half of the richness in year-old beech leaf litter.

Contrary to our hypothesis, endophyte community richness and abundance did not differ across the abiotic stress gradient (Table 2). This suggests that, unlike mycorrhizal fungi (Jacobson, 1997), the benefits of possessing a functionally diverse latent-saprophyte endophytic community outweigh the costs to the living plants. Furthermore, the functional diversity of eastern and western communities did not differ in the proportion of decomposition-capable isolates, the mean percent mass loss of all isolates, or the richness and abundance proportions of the endophyte community that were also in active litter. These asymptomatic latent saprophytes appear to be of sufficient functional importance that plants support them in their living tissues regardless of the abiotic stress they are experiencing. Our results corroborate a recent study that found differences in community composition across the gradient, but functional performance (litter decomposition rates) did not differ, nor did fungal diversity and richness amongst these surface litter communities (Logan et al., 2021).

Based on our study, we propose that the need to efficiently recycle nutrients is why diverse foliar endophyte communities are present in plants in extreme environments (e.g., Rosa et al., 2009; Massimo et al., 2015; González-Menéndez et al., 2018). While individual species may confer advantages to the living plant, our data strongly supports the hypothesis that plants tolerate diverse communities of latent saprophytes throughout their lifetime because of the role that the community plays at senescence. By hosting latent saprophytes as endophytic communities, living tillers are pre-inoculated with a functionally diverse saprophytic community that can immediately commence decomposition in response to frequent NRM and rain events that moisten standing litter (Jacobson et al., 2015; Evans et al., 2020; Logan et al., 2021). Such a strategy may be particularly important to dryland perennial plants because inorganic soil nitrogen and phosphorus are often limiting (Ronca et al., 2015), and therefore plant organic matter is a critical nutrient source. Nutrient islands beneath perennial plants are a well-documented dryland phenomenon, where N & P levels may be 3–10 times higher than in the soil matrix between plants (Klopatek and Stock, 1994; Abrams et al., 1997; André et al., 1997; Throop et al., 2020). Plant morphological traits, such as the caespitose stem structure of bunch grasses (see Figure 1A), provide the structural support for these islands to the benefit of perennial plants. Likewise, we propose that the ability of perennial plants to accept/recruit and maintain specific latent saprophytes in their living tissue, in preparation for senescence, is an essential trait that contributes to efficient mineralization and retention in these nutrient islands.

Foliar endophyte communities in perennial plants in more mesic habitats are also composed of complex assemblages of latent saprophytes, and a host of fungi whose functional attributes are not yet known (Arnold, 2007; Porras-Alfaro and Bayman, 2011). Given the large proportion of latent saprophytes in beech leaves that are present a year after senescence (Guerreiro et al., 2018), endophytes may play a similar recycling role in the root zone of forest trees. It may even be important in mesic nutrient-rich prairies where space in the root zone is at a premium and thus nutrient retention by individual plants is important. Indeed caespitose grasses, such as *Andropogon gerardii* (big blue stem) and *Sorghastrum nutans* (Indian grass), but not rhizomatous grasses, have “nutrient islands” of enriched N & P in their root zones (Derner and Briske, 2001). It is worth noting that fungal communities on standing big bluestem litter are as responsive to NRM events (dew, fog and high humidity) as those in the NSS, and that “wet events” (including rainfall) occur 77% of the time in temperate prairie habitats (Evans et al., 2020). Likewise, fungi on standing litter of little bluestem species in a longleaf pine savanna ecosystem account for 55–65% of carbon loss (Lodato et al., 2021), and all dominant taxa are known endophytes. The ability to tolerate communities of asymptomatic latent saprophytes as endophytes in living tissue, so that they can initiate *in situ* nutrient cycling immediately upon senescence, may well be an essential trait of any perennial plant facing nutrient limitations.

## Data Availability Statement

The raw data supporting the conclusions of this article will be made available by the authors, without undue reservation.

## Author Contributions

AW and KJ conceived of the study and wrote the manuscript. KJ, PJ, MS, JL, and SE conducted field work. AW, AD, KJ, JL, and SE did lab work and data analysis. All authors edited the manuscript and approved the submitted version.

## Conflict of Interest

The authors declare that the research was conducted in the absence of any commercial or financial relationships that could be construed as a potential conflict of interest.

## Footnotes

1 http://ncbi.nlm.nih.gov/

2 http://www.cbs.knaw.nl/

3 http://www.sasscalweathernet.org/
